# Physical Fitness of the Older Adult Community Living in Leiria, Portugal

**DOI:** 10.3390/epidemiologia5030023

**Published:** 2024-07-02

**Authors:** Filipe Rodrigues, Diogo Monteiro, Rui Matos, Miguel Jacinto, Raúl Antunes, Paulo Gomes, Nuno Amaro

**Affiliations:** 1 ESECS—Polytechnic of Leiria, 2411-901 Leiria, Portugal; diogo.monteiro@ipleiria.pt (D.M.); rui.matos@ipleiria.pt (R.M.); miguel.s.jacinto@ipleiria.pt (M.J.); raul.antunes@ipleiria.pt (R.A.); nuno.amaro@ipleiria.pt (N.A.); 2 Research Center in Sport, Health and Human Development (CIDESD), 5000-558 Vila Real, Portugal; 3 Municipality of Leiria, 2414-006 Leiria, Portugal; pgomes@cm-leiria.pt

**Keywords:** strength, cardiorespiratory fitness, flexibility, balance, body composition, elderly

## Abstract

This study aimed to achieve two primary objectives. Firstly, to compare physical fitness levels based on sex, age groups, and body composition. Secondly, to examine physical fitness of the older adults against criterion-referenced fitness standard values using a representative sample from the district of Leiria in Portugal, a region undergoing exponential population growth, including a rise in the elderly population. Data from cross-sectional assessments of the Portuguese older adult community living in the Municipality of Leiria from 2014 to 2022 were collected. This study comprised a sample of 664 community-dwelling older adults (female = 480; male = 184) aged between 60 and 90 years (M = 70.34; SD = 12.67). Participants had a body mass ranging from 45 kg to 122 kg (M = 71.48; SD = 12.34) and a height ranging from 1.30 m to 1.89 m (M = 1.59; SD = 0.08). The Senior Fitness Test battery was used to evaluate parameters of physical fitness, body mass index was calculated, and abdominal circumference was measured. Females displayed a tendency to greater physical fitness compared to males, specifically in terms of lower and upper body flexibility compared to older male adults (*p* < 0.05). In addition, sexagenarian and older adults with normal weight tended to display greater physical fitness compared to septuagenarian and octogenarian, and overweight adults and those with obesity (*p* < 0.05), respectively. Information concerning physical fitness is crucial for guiding policymakers and other stakeholders in Leiria for the promotion of healthy aging among the older adult community. Furthermore, this study has established the preliminary reference standards for physical fitness, serving as a tool to promote healthy aging among Portuguese older adults residing in Leiria.

## 1. Introduction

The investment in the study of aging was a logical response to the challenges posed by an aging population at the beginning of the 21st century, such as the increased expenditure on health and long-term care and the potential problems with old-age income security [1]. The global trend of aging is undeniable, with the percentage of individuals over 65 years being the fastest-growing segment, and 125 million people already aged 80 years or older [2]. This demographic shift has resulted from an increased average life expectancy and decreased birth rates, leading to an exponential rise in the elderly population compared to younger cohorts [3]. According to the United Nations projections, the elderly population will reach 2 billion by 2050, bringing significant economic, social, and healthcare challenges to most countries [4]. According to data from the 2021 Census conducted by the Portuguese National Institute of Statistics [5], the number of residents in Portugal aged over 65 was 2,423,639, representing approximately 23.4% of the total population. Considering these data, it is comprehensible to speculate that the elderly population in Portugal will increase dramatically in the coming decades, making the study of aging a highly relevant contemporary topic [6,7,8].

There are several physical aspects of aging that must be considered. Generally, aging is associated with an increased risk of non-communicable diseases, frailty, disability, and early all-cause mortality, all of which can represent a significant burden on national healthcare systems [8,9]. On an individual level, several structural and functional changes occur during the aging process, such as loss of walking ability, muscle strength, balance, and flexibility. As individuals age, physical fitness levels decrease uniformly with advancing age [10,11]. The maintenance of flexibility, production of force and power, and coordination depend on physical fitness levels during previous life stages, such as adulthood. Physical fitness may be indicative of quality of life, as sufficient muscle strength, cardiorespiratory capacity, agility, and dynamic balance are all important contributors to reduced risk of falls, greater autonomy, and independence [12,13]. Hence, it is paramount to define the functional fitness level of older adults and determine how existing community programs that encompass physical activity may increase functional fitness and reduce fall risk and fall-related injuries, thereby increasing quality of life. Given the evolving characteristics and diversity of the increasing older adult population in Portugal, specifically in the center region, along with limited or nonexistent data on healthy aging, it is crucial to have monitoring systems capable of assessing trends and establishing the percentages of older adults achieving the standard values for aging-related aspects, including functional fitness [6]. Information regarding the changes in functional fitness is crucial for guiding policymakers and other stakeholders in achieving the goals outlined in the international agenda [14]. Specifically, the aim is to promote quality of life among the elderly population, creating conditions for autonomy and independence for those living in the community, thereby reducing the low levels of physical fitness associated with the high risk of falling, low quality of life, and premature death associated with several comorbidities.

This study had two main objectives. First, to compare physical fitness levels based on sex, age groups, and body composition. Second, to compare physical fitness levels with normative reference values, using a representative sample from the district of Leiria, a region experiencing exponential population growth, including an increase in the elderly population [5]. Indeed, the statistical data indicate a 15.4% increase in people over 65 years old in the coastal area of Portugal, making Leiria a prominent region for the study of physical fitness in the elderly. Considering the previous literature [6,7,8], it was hypothesized that there would be significant differences in physical fitness levels between the different sexes, age groups, and body compositions. Also, it was speculated that the physical fitness levels of the elderly population in Leiria would be similar to those reported in the previous national studies and similar to the cutoff values reported in the literature [7,8].

## 2. Materials and Methods

### 2.1. Participants

This study comprised a sample of 664 community-dwelling older adults (female = 480; male = 184) aged between 60 and 90 years (M = 70.34; SD = 12.67). Participants had a body mass ranging from 45 kg to 122 kg (M = 71.48; SD = 12.34) and a height ranging from 1.30 m to 1.89 m (M = 1.59; SD = 0.08). In terms of age groups, 323 participants were aged between 60 and 69 years (i.e., sexagenarians), 286 were aged between 70 and 79 years (i.e., septuagenarians), and 55 were aged between 80 and 89 years (i.e., octogenarians). Only one person was nonagenarian. Regarding body mass index (BMI), 173 participants (26.1%) were of normal weight, 284 participants (42.8%) were overweight, and 207 participants (31.2%) had obesity, according to the following ranges from the World Health Organization (WHO): normal weight = BMI = 15–25 kg/m^2^; overweight = BMI = 25.01–29.99 kg/m^2^; and obesity = BMI > 30 kg/m^2^.

Participants were included in the study if they met the following inclusion criteria: being an older adult aged 60 to 90 years living in the community of Leiria; not suffering from severe cognitive impairment having no medical contraindications for performing physical fitness and body composition tests; participating voluntarily; and being able to move independently without using a manual mobility device (e.g., cane or walker).

### 2.2. Data Collection Procedures

Before beginning the data collection, this study was reviewed and approved by the Ethics Committee of the Polytechnic Institute of Leiria (omitted for review purposes). The data collection procedure consisted of two main phases. In the first phase, the coordination of the Viver Ativo Program (Municipality of Leiria, 2023), a community initiative led by the Municipality of Leiria promoting physical exercise for the local older adult community, is contacted for the study team to present the objectives of the present study. The Viver Ativo program was contacted since it is the largest physical activity program for seniors in the Leiria district, with an annual increase in new participants. Therefore, it is understandable that researchers contacted this program in order to obtain sufficient data to ensure the necessary sample size for representativeness. The Viver Ativo Program regularly recruits participants through flyers, newspapers, and social media communications. The participants are recruited equally with the support of the local councils to ensure greater proximity to the target population. Thus, sample recruitment can be both random and by convenience. When the participants sign up for the Viver Ativo program, they sign an informed consent for participation. They also complete a sociodemographic questionnaire, and all the participants are assessed before participating in the different exercise programs. Afterwards, they are informed about the possibility of participating in scientific research by providing consent of non-personal data for research purposes. Information about the study objectives is then provided to all potential participants to ensure voluntary participation and address any questions. Participants are thoroughly informed about the voluntary nature of their participation and assured of their right to withdraw from the study at any time without any negative consequences. In the second phase, following institutional approval, the technical team is briefed on the objectives of the study and asked for their support in data collection, as they are responsible for conducting the annual initial evaluations of the new Viver Ativo participants at the start of the program in September. In line with this study, the technical team was requested to assist in selecting the data according to the inclusion criteria.

### 2.3. Instruments

To collect demographic data for the sample characterization, a questionnaire was used including the following elements: age and sex. The Viver Ativo Program collects other data, such as marital status and academic qualifications, but these were not considered for this study as they are not relevant to the objectives of the current study.

#### 2.3.1. Lower Body Muscle Endurance

The 30 s chair stand test, included in the Senior Fitness Test battery [15], was used to assess lower body muscle endurance. During the test, the participant would start seated in a chair, crossing their arms, and would be instructed to repeatedly stand up and sit down for thirty seconds, aiming to complete as many repetitions as possible. The following tests were individually explained to the participants of this study. Additionally, the researcher demonstrated the correct execution of the tests, enhancing each participant’s understanding. Participants were given two attempts for each test, and the best score was used for analysis.

#### 2.3.2. Upper Body Muscle Strength

The 30 s arm curl test, also part of the Senior Fitness Test battery [15], was used to assess upper body muscle strength. The participant would sit in a chair, holding a dumbbell in their dominant hand, starting from full elbow extension. The participant would then be instructed to perform as many elbow curls as possible in thirty seconds. A 2 kg dumbbell was used for female participants and a 3 kg dumbbell for male participants.

#### 2.3.3. Agility and Dynamic Balance

The timed up-and-go test, part of the Senior Fitness Test battery [15], was used to assess dynamic balance and agility. The test involves walking a straight distance, starting from a seated position in a chair, walking to a cone placed 2.44 m away, circling it, and returning to the initial chair. Participants were instructed to stand up, walk quickly without running, circle the cone, return to the chair, and sit down again. An initial measurement was taken for familiarization, and the completion time was then recorded.

#### 2.3.4. Flexibility

The chair sit-and-reach test, included in the Senior Fitness Test battery [15], was used to assess lower body flexibility. During the test, the participant would sit in a chair, extend their dominant leg forward with the foot flexed, and reach forward toward their toes with one hand over the other, maintaining full knee extension. The distance reached (or not reached) was recorded. Additionally, the back scratch test, also part of the Senior Fitness Test battery [15], was used to assess upper body flexibility. The participant would stand with their feet shoulder-width apart, extending one arm over the shoulder while keeping the other arm behind their back, and then attempt to touch the opposite hand. The distance reached (or not reached) was recorded.

#### 2.3.5. Body Mass Index

Height was measured using a portable Seca 213 stadiometer with an integrated level (GmbH & Co. KG, Hamburg, Germany). Participants stood with their back against a wall where the stadiometer was fixed, ensuring the back of their head, shoulders, and buttocks touched the wall. With their weight evenly distributed on both feet, participants looked straight ahead, inhaled, and held their breath. The stadiometer’s measuring arm was lowered to the top of the head, and the measurement was recorded. Weight was measured using a portable Seca 813 digital balance (GmbH & Co. KG, Hamburg, Germany) placed on a level and stable surface. Participants were weighed without shoes and coats, retaining other clothing. They were instructed to stand on the scale with feet positioned according to the scale’s reference, looking straight ahead and remaining still during the measurement. BMI was calculated using the following formula: BMI (kg/m^2^) = weight (kg)/height^2^ (m).

#### 2.3.6. Waist Circumference

Waist circumference was measured using a measuring tape. Participants stood upright with a relaxed abdomen, arms naturally at their sides, palms facing inward, head straight, and feet together, evenly distributing their weight. After removing their coats and lifting their shirts to expose the waist and hip area, the tape was placed around the waist at the level of the navel. Participants were then instructed to exhale normally, and the measurement was taken at the end of the exhalation. This procedure was repeated twice, and the final value was calculated as the average of the two measurements obtained.

### 2.4. Data Analysis

All statistical analyses were conducted using IBM SPSS Statistics 29.0 (IBM, Armonk, NY, USA). Descriptive statistics, including mean, standard deviation, skewness, and kurtosis, were calculated. Additionally, the Kolmogorov–Smirnov test was applied to assess normality within each group. The results indicated a normal distribution (*p* > 0.05). Thus, the independent samples *t*-test was employed to examine potential differences based on sex. Homogeneity was assessed using Levene’s test, considering equal variances if the significance value was less than 0.05. Significant differences were determined if the *t*-test yielded a *p*-value < 0.05. Cohen’s d [16] was calculated to ascertain effect sizes for the *t*-test analysis, categorized as trivial (0–0.19), small (0.2–0.49), medium (0.5–0.79), and large (0.80 or greater). For comparisons based on age groups and body mass index, one-way analysis of variance was performed. Significant differences were identified if the test produced a *p*-value < 0.05. Post hoc tests, adjusted with the Bonferroni method, were conducted to explore group comparisons. Partial eta squared was computed to evaluate the effect size for comparisons among multiple groups, with reference values for small (0.01), medium (0.06), and large (0.14) effects. Mean and standard deviation for age groups (60–64, 65–69, 70–74, 75–79, 80–84, 85–90 years) and sex were calculated for the 30 s chair stand, arm curl, chair sit-and-reach, back scratch, 8 foot up-and-go, and 2 min step tests. Afterwards, we compared the individual scores against the criterion-referenced fitness standard proposed by Rikli and Jones [15], and the percentage of participants who succeeded in meeting the national standard were calculated.

## 3. Results

In Table 1, we can observe the results of the descriptive and inferential statistics of physical fitness levels according to sex. Statistically significant differences were found in both flexibility tests (*p* < 0.001). The results indicate that females exhibited higher levels of flexibility in the upper and lower limbs compared to males. As for the remaining physical fitness and body composition tests, no statistically significant differences were found. In terms of effect size, these were moderate (d = 0.652–0.749).

In Table 2, we observe the results of descriptive and inferential statistics regarding the physical fitness levels across the different age groups. Statistically significant differences were found in both flexibility tests, the timed up-and-go test, as well as in the cardiorespiratory fitness test (*p* < 0.001). The results indicate that the group aged 60 to 69 years exhibited higher levels of flexibility in both upper and lower limbs compared to the groups aged 70 to 79 years and 80 to 89 years. However, the group aged 60 to 69 years showed lower levels of cardiorespiratory capacity, as measured by the 2 min step test, compared to the group aged 70 to 79 years. No statistically significant differences were found in the remaining physical fitness tests and body composition. In terms of effect size, these differences were small (d = 0.009–0.025).

Regarding comparisons between the groups based on body mass index, the group of individuals with normal weight showed statistically significant differences compared to the group of individuals with obesity in all the tests (see Table 3). However, the group of individuals with normal weight only showed statistically significant differences in the back scratch test and waist circumference compared to the group of overweight individuals. There were statistically significant differences between the group of overweight individuals and those with obesity in several physical fitness tests (*p* < 0.05). Regarding the significant differences between the groups, the group of individuals with normal weight showed better levels of physical fitness compared to the group of overweight or morbidly obese individuals. In terms of effect size, these were moderate (η2p = 0.016–0.061).

Table 4 presents the average functional fitness test scores according to sex and age groups for Portuguese older adults living in Leiria. Males performed better than females on some tests within the early age groups; however, in the later age groups, the females tended to display better physical fitness. Females performed better on the two flexibility tests (i.e., chair sit-and-reach and back scratch), and the differences between men and women did reach significance in several age groups.

Around half of the Portuguese older adult population of Leiria met the criterion-referenced fitness standards associated with independent functioning for the 30 s chair stand (56.0%) and arm curl (58.9%) tests. However, less than 30% met the recommended criteria for the other functional tests (see Table 5). A higher percentage of Portuguese females met the standards for the arm curl, 6 min walk, and 8 ft up-and-go tests. In addition, more older female adults tended to meet the cutoffs for the sit-and-reach and the back scratch tests (*p* < 0.05). Overall, older female adults displayed a tendency to greater physical fitness compared to older male adults based on the criterion-referenced standards.

## 4. Discussion

This study had two main objectives. First, to compare the physical fitness levels based on sex, age groups, and body composition. Second, to compare the physical fitness levels with the normative reference values, using a representative sample from the district of Leiria. The results from this investigation provide, for the first time, trends and values for the functional fitness tests of older Portuguese adults living in Leiria. Overall, older adult females tended to display greater physical fitness compared to their male counterparts. Current findings suggest that physical fitness levels for both the males and females tend to be lower than the criterion-referenced fitness values [15] and in the observational cross-sectional studies [6,7,17]. However, females tended to display higher levels of upper and lower body flexibility. Regarding the risk of falling, based on the timed up-and-go test, which is a reliable measure of agility and dynamic balance associated to body stability, both male and female older adults were at risk, with only 22.3% of males and 31.5% of females meeting the criteria.

As part of the aging process, recent reports have advocated for older adults to adopt a healthy lifestyle, where physical activity and functional fitness are the cornerstones for postponing functional declines, such as the increased risk of chronic diseases and falling, which are major international concerns today. Given their widespread use, the senior fitness tests provide a means not only to assess older adults’ physical fitness levels but also to monitor trends in regional and national representative samples, as described in the recent literature. Overall findings indicate that older Portuguese adults living in Leiria have declined in physical fitness tests compared to nationwide reports. Specifically, current results showed that older adult females tended to display greater physical fitness compared to males, which contradicts previous trends. We hypothesize that females tend to be more physically active compared to males in the western region of Portugal, possibly due to greater involvement in household and professional activities which has been reported in other studies [18,19]. However, this is purely speculative and warrants further research.

The mean scores did not decrease significantly across the age groups. While the subsamples were heterogeneous, we observed that in almost all fitness tests, the age group with the highest average score was 75–79 years in both the male and female groups, compared to other subgroups, mainly in the muscular endurance tests, which could be attributed to various reasons, such as sample heterogeneity which has been highlighted. For example, there were less participants in this study aged 75–79 years compared to participants aged 65–69 years. The literature describes aging as negatively impacting physical fitness and, at least in Portugal, older adults tend to lose their functional capacity [6,7,17]. It is also expected that the number of participants per subgroup decreases with increasing age, considering the average life expectancy and the life expectancy at age 65. These two points may be related or interdependent. However, considering that the WHO [2] has highlighted that the average life expectancy and the life expectancy at age 65 are different, with the latter representing more years of life than the former, thus there is some exploratory evidence that the people who were assessed at the beginning of the Viver Ativo program and who were already over 70 or 75 years old had higher levels of physical fitness compared to those who were 60 or 65 years old. Nonetheless, overall, this sample of older adults living in the community in Leiria displayed the tendency to a decline in physical fitness similar to those reported in nationwide studies [6,7,8,17].

The results of the timed up-and-go test are particularly notable, revealing that, on average, more than 50% of the elderly population living in Leiria, regardless of sex or age group, exhibited an increased risk of falling. These findings contrast with a previous study [20], where the older adults living in the community displayed relatively high levels of agility and balance, physical components that were associated with this test. This finding is of the utmost importance and should be addressed to mitigate the risk of falls among the elderly in Leiria, thereby promoting greater independence and autonomy.

The data from this exploratory study clearly demonstrate the need for the implementation of physical activity programs to significantly enhance the physical fitness of the elderly population. Less than 50% of participants from this representative sample in Leiria exhibited physical fitness levels above the reference criteria. Indeed, the benefits of physical activity on the various important quality of life indicators for the elderly are well documented [21,22]. In response to this need, the Leiria City Council established the Viver Ativo Program, a physical activity initiative open to the elderly community, aimed at mitigating the adverse effects of aging on physical fitness. The program has since attracted dozens of participants and has been expanding in both the number of participants and the variety of activities offered, including hydro-gymnastics classes, group classes focused on improving muscular and cardiorespiratory endurance, as well as a dedicated space for strength training [23]. This study seeks to continue monitoring the physical fitness of the elderly in this community to identify the most effective physical practices for improving their fitness and to determine how more people can access this community physical activity program. Thus, this study serves as a starting point, clearly highlighting the importance of scientific research in developing strategies and policies to promote physical activity within the community.

### Limitations and Agenda for Future Research

One of the major strengths of this investigation lies in its focus on the first analysis of regional data, as opposed to studies that have considered Portugal as a whole. Additionally, the sample is representative, and the data were collected using a rigorous methodology, with tests administered by professionals experienced in the assessment and prescription of exercise for older adults. Another important aspect is the use of the Senior Fitness Test battery, which was developed and validated to assess the functional fitness of community-residing older adults in a field setting [15]. The tracking of trends and the establishment of percentages of the older adults achieving fitness standard values in these tests hold significant value not only for policymakers but also for researchers, healthcare providers, and other stakeholders directly or indirectly involved in promoting healthy aging in the Leiria region.

Current findings are also limited by several factors such as sample characteristics and study design. Although the present study had a reasonable sample size and included participants up to 80 years old, heterogeneity of the subsamples (age groups) limits interpretation and thus more data are needed. Additionally, data collected by a municipality program were used, which leads to convenience sampling procedures. It is also important to highlight the heterogeneity of the sample per year, which limits the longitudinal analysis of the physical fitness levels of the elderly residents in Leiria and thus hinders the identification of trends as performed in previous studies [7,8]. These gaps are intended to be addressed in future studies. Regarding the second potential limitation, the cross-sectional design is inadequate for the capture of the temporal relations that occur throughout older adult stages, which precludes the inference of causality between physical activity and functional ability. In fact, the authors did not assess the previous physical activity before signing up to the community program. Likewise, considering that the initial assessment data were used, it would make sense to reassess the participants of this study now that they are physically active and enrolled in a community exercise program to analyze changes in their physical fitness levels.

## 5. Conclusions

This study provides preliminary evidence of population-specific and relevant data for older Portuguese adults living in Leiria. Muscle strength, aerobic endurance, flexibility, agility, dynamic balance, and body composition were found to have declined with age, and females generally performed better on tests of functional fitness than males. Furthermore, and notably, older adults of both sexes tended to display an elevated risk of falling, due to the low agility and dynamic balance capacities measured using the timed up-and-go test. Therefore, any intervention in older adults should consider the loss of functionality and differences between women and men in fitness performance. More research, particularly longitudinal studies including adults over 60, 70, and 80 years of age, is needed for a more thorough understanding of these relations.

Given the heterogeneity of the older adult population and the limited or non-existent data on the healthy aging process, it is crucial to have monitoring systems that can assess trends related to aging, such as functional fitness. In line with the goals set forth by international agendas, this investigation provides regional data for functional fitness tests of older Portuguese adults living in Leiria for the first time. The information presented by this investigation will offer a means to compare the efficacy of healthcare policies applied in future time periods and how they impact older adults’ functional fitness.

## Figures and Tables

**Table 1:** Descriptive and inferential analysis according to sex.

Variables	Male		Female		Levene Test	*p*	t	*p*	d
	M	SD	M	SD					
30 s Sit and stand	14.50	4.00	14.50	3.80	0.097	0.756	0.003	0.499	-
30 s arm curl	17.39	4.75	17.10	4.35	2.702	0.101	−0.745	0.228	-
Sit and reach	−4.02	10.62	2.10	8.85	14.797	<0.001	6.942	<0.001	0.652
Back scratch	−18.23	14.52	−9.12	11.14	14.732	<0.001	7.688	<0.001	0.749
Timed up and go	6.41	2.38	6.43	2.47	0.346	0.556	0.071	0.472	-
2-min step test	115.15	72.30	106.66	60.75	14.067	<0.001	−1.413	0.064	-
Waist circumference	100.59	12.17	96.75	38.53	0.405	0.525	−1.326	0.093	-

Notes: M = mean; SD = standard deviation; *p* = significance level; d = effect size.

**Table 2:** Descriptive and inferential analysis according to age groups.

Variables	60–69 Years		70–79 Years		80–89 Years		F	*p*	η^2^p	Comparisons between Groups
	M	SD	M	SD	M	SD				
30 s Sit and stand	14.40	3.53	14.73	4.20	13.85	3.78	-	-	-	-
30 s arm curl	17.14	4.37	17.37	4.65	16.42	4.03	-	-	-	-
Sit and reach	1.34	9.07	−0.35	10.57	−1.23	8.84	3.15	0.03	0.009	1 ≠ 2; 1 ≠ 3
Back scratch	−9.79	11.20	−12.88	14.00	−16.09	13.69	8.17	<0.001	0.024	1 ≠ 2; 1 ≠ 3
Timed up and go	6.22	2.47	6.42	2.24	7.66	2.92	8.38	<0.001	0.025	1 ≠ 3; 2 ≠ 3
2-min step test	101.87	60.30	117.62	67.48	106.20	64.82	4.67	0.010	0.014	1 ≠ 2
Waist circumference	96.57	11.71	99.38	49.09	96.93	12.23	-	-	-	-

Notes: M = mean; SD = standard deviation; *p* = significance level; F = F-test results; *p* = significance; η2p = partial eta squared; ns = no significant differences detected.

**Table 3:** Descriptive and inferential analysis according to body mass index.

Variables	Normal Weight		Overweight		Obese		F	*p*	η2p	Comparisons between Groups
	M	SD	M	SD	M	SD				
30 s Sit and stand	15.38	4.16	14.82	3.63	13.32	3.62	15.77	<0.001	0.046	1 ≠ 3; 2 ≠ 3
30 s arm curl	17.82	4.65	17.38	4.48	16.38	4.19	5.401	<0.05	0.016	1 ≠ 3; 2 ≠ 3
Sit and reach	2.02	9.25	0.68	8.91	−1.33	10.98	5.84	<0.005	0.017	1 ≠ 3
Back scratch	−5.37	11.72	−12.43	11.99	−15.81	12.87	35.49	<0.001	0.097	1 ≠ 2 ≠ 3
Timed up and go	5.81	1.53	6.28	2.57	7.14	2.71	15.35	<0.001	0.044	1 ≠ 3; 2 ≠ 3
2-min step test	124.20	65.17	115.63	63.44	87.23	58.86	19.26	<0.001	0.055	1 ≠ 3; 2 ≠ 3
Waist circumference	85.67	8.16	98.06	48.44	107.62	9.70	21.62	<0.001	0.061	1 ≠ 2 ≠ 3

Notes: M = mean; SD = standard deviation; *p* = significance level; F = F-test results; *p* = significance; η2p = partial eta squared; ns = no significant differences detected.

**Table 4:** Fitness means and SD according to sex and age group.

Variables	All		60–64		65–69		70–74		75–79		80–84		85–89	
	M	SD	M	SD	M	SD	M	SD	M	SD	M	SD	M	SD
30 s Sit and stand														
Male	14.50	4.00	14.29	2.02	14.53	3.36	14.23	4.04	15.04	5.05	14.00	4.33	13.80	2.86
Female	14.51	3.80	14.36	3.35	14.40	3.86	15.13	3.97	14.17	3.85	13.95	3.85	13.75	2.63
30 s arm curl														
Male	17.39	4.75	18.41	5.96	17.29	4.39	17.04	4.67	17.71	5.07	17.47	4.36	15.20	3.42
Female	17.12	4.34	17.29	4.17	16.85	4.30	17.87	4.58	16.50	4.00	16.33	4.00	16.75	0.96
Sit and reach														
Male	−4.02	10.62	−3.27	10.56	−2.00	10.26	−5.23	10.93	−4.41	11.43	−4.87	8.59	−8.00	9.92
Female	2.10	8.86	1.85	8.89	2.60 *	8.22	2.31 *	9.43	1.39 *	8.01	0.68 *	8.01	6.25 *	7.50
Back scratch														
Male	−18.23	14.52	−14.00	9.98	−18.16	13.77	−19.20	14.83	−17.55	15.59	−23.73	17.89	−13.60	8.73
Female	−9.09	11.13	−7.79	10.40	−8.01 *	9.56	−9.38 *	12.83	−11.10 *	10.29	−14.29 *	10.29	−2.75	13.20
Timed up and go														
Male	6.41	2.38	5.96	1.35	6.16	1.48	6.52	3.47	6.43	2.11	7.38	2.40	6.29	0.95
Female	6.40	2.40	6.40	3.77	6.13	1.47	6.31	1.46	6.55	2.79	7.66	2.79	7.53	1.39
2-min step test														
Male	115.15	72.30	80.53	49.91	115.47	72.55	123.01	77.21	120.54	74.89	118.33	69.30	89.80	56.57
Female	106.80	60.73	96.54	55.13	103.65	59.97	115.65	62.39	115.08	66.26	103.97	66.26	114.50	61.14
Waist circumference														
Male	100.59	12.17	103.44	11.05	99.51	13.10	101.26	12.03	98.52	13.18	105.27	7.65	100.50	4.72
Female	96.75	38.53	95.77 *	10.45	95.40 *	11.85	101.93	74.37	94.12 *	12.41	93.62 *	13.15	86.00 *	8.83

Notes: M = mean; SD = standard deviation; * Significantly different from females, *p* < 0.05.

**Table 5:** Percentage of participants meeting sex and age group-specific fitness standards associated with independent functioning.

Variables	All	60–64	65–69	70–74	75–79	80–84	85–89
30 s Sit and stand							
Male	38.0	5.9	32.7	30.0	50.0	66.7	80.0
Female	41.0	33.6	30.7	49.2	54.4	53.3	75.0
30 s arm curl							
Male	46.7	23.5	38.8	42.0	58.3	66.7	80.0
Female	49.4	44.9	42.0	55.8	55.8	60.0	100.0
Sit and reach							
Male	57.1	52.9	63.3	54.0	54.2	60.0	60.0
Female	78.3	80.4	77.3	72.5	85.3	83.3	100.0
Back scratch							
Male	22.3	23.5	24.5	18.0	22.9	20.0	40.0
Female	31.5	32.7	34.0	29.2	32.4	23.3	25.0
Timed up and go							
Male	35.9	17.6	20.4	40.0	47.9	40.0	80.0
Female	31.9	22.4	30.0	35.0	47.1	30.0	25.0
2-min step test							
Male	50.0	17.6	46.9	58.0	54.2	60.0	40.0
Female	50.6	39.3	44.7	60	63.2	53.3	75.0

## Data Availability

The data utilized in this study were obtained under a specific license exclusively for the purposes of this research. The data supporting the findings of this study are not publicly available but can be requested and accessed upon reasonable inquiry, subject to permission from the City Council of Leiria.
